# An Overview of the Relevance of IgG4 Antibodies in Allergic Disease with a Focus on Food Allergens

**DOI:** 10.3390/children8050418

**Published:** 2021-05-20

**Authors:** Thomas A. E. Platts-Mills, Behnam Keshavarz, Jeffrey M. Wilson, Rung-chi Li, Peter W. Heymann, Diane R. Gold, Emily C. McGowan, Elizabeth A. Erwin

**Affiliations:** 1 Division of Allergy & Clinical Immunology, Department of Medicine, University of Virginia, Charlottesville, VA 22908, USA; bk4fy@virginia.edu (B.K.); jmw2gc@virginia.edu (J.M.W.); rl6sf@virginia.edu (R.-c.L.); pwh5a@virginia.edu (P.W.H.); ekc5v@virginia.edu (E.C.M.); 2 Department of Environmental Health, Harvard T.H. Chan School of Public Health, Harvard University, Boston, MA 02115, USA; diane.gold@channing.harvard.edu; 3 Channing Division of Network Medicine, Brigham and Women’s Hospital, Harvard Medical School, Boston, MA 02115, USA; 4 Division of Allergy and Immunology, Nationwide Children’s Hospital, Columbus, OH 43205, USA; Elizabeth.Erwin@nationwidechildrens.org

**Keywords:** food allergy, IgG4 antibody, Fab-arm exchange, eosinophilic esophagitis, milk, wheat, food processing

## Abstract

Antibodies of the IgG4 isotype are strongly associated with allergic disease but have several properties such as not precipitating with allergens, not activating complement and poor binding to Fcγ receptors that argue against a pro-inflammatory role. In keeping with that, IgG4 antibodies are a striking feature of the response to immunotherapy. In two naturally occurring situations IgG4 antibodies are common with low or absent IgE antibodies. The first example is children raised in a house with a cat and the second is eosinophilic esophagitis (EoE). In many population-based cohorts, the ownership of a cat in early childhood is associated with a decreased prevalence of a cat allergy at age 10. The second example (i.e., EoE) is a novel form of food allergy that is not mediated by IgE and is related to consuming cow’s milk or wheat. In EoE, patients have IgG4 to milk proteins in high > 10 µg/mL or very high > 100 µg/mL titers. Enigmatically these patients are found to have deposits of IgG4 in the wall of their inflamed esophagus. The factors that have given rise to EoE remain unclear; however, changes in food processing over the past 50 years, particularly ultra-heat treatment and the high pressure homogenization of milk, represent a logical hypothesis.

## 1. Introduction

The IgG4 isotype was first described together with the other two minor sub-classes (i.e., IgG2, IgG3) that make up IgG prior to 1970 [1,2]. The total quantities of IgG4 in the circulation are lower than the main isotypes (i.e., IgG1, IgM, IgA) but similar to IgD and far higher than IgE [3,4]. Interestingly each of the isotypes with low mean quantities in the serum have a strikingly wide dynamic range. It was soon recognized that IgG4 molecules have several interesting properties. Firstly, Kunkel reported that a significant proportion, up to 10%, of serum IgG4 was circulating in the form of a monomer, i.e., one heavy chain with one light chain [1]. In addition, he identified a subject who had a genetic difference of one amino acid in the hinge region who had no monomers of IgG4 in his circulation. This finding clearly implied that minor changes in the hinge region could influence the stability of the dimer form of IgG4. Other features that were identified early included that this isotype does not activate a complement and has a relatively poor ability to bind to Fc gamma receptors except for inhibitory receptor FcγRIIB [5]. Finally, it became clear that IgG4 antibodies not only do not make precipitin reactions in a gel but can also inhibit the ability of IgG or other antibodies to precipitate with a relevant protein [6]. At this point there was already evidence that IgG4 antibody responses were related to allergic disease; however, they were increasingly considered to have a role in controlling allergic inflammation.

The reputation that IgG4 antibodies could be anti-inflammatory or tolerogenic was enhanced by studies showing increases in a specific IgG4 in response to immunotherapy with pollen or mite extracts [7,8]. In addition, detailed studies on IgG4 antibodies to venom proteins were carried out both during venom immunotherapy (IT) and in beekeepers who “self-induced” tolerance by receiving multiple bee stings during the summer [9]. Taken together, the evidence about the properties of IgG4 and the clinical data provided a convincing body of evidence that antibodies of the IgG4 isotype should be regarded as an element of tolerance to allergens.

## 2. Fab-Arm Exchange

**Figure 1:**
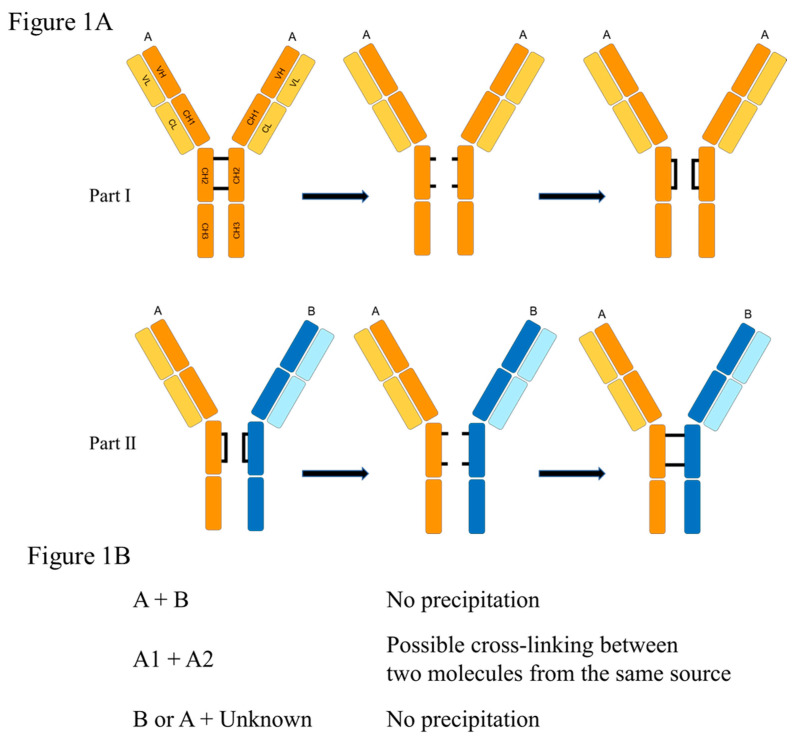
(**A**) Fab-arm exchange in IgG4 molecule. The separation of the two arms depends on the ability to create stable disulfide bonds within each arm (Part I); the recombination with other arms is random (Part II). (**B**) We propose that the quantities of the three forms of the recombined molecules depends on the quantity of specific IgG4 antibodies in the circulation.

Twenty years ago, Aalberse and his colleagues in Amsterdam presented evidence that some of the circulating allergen-specific IgG4 molecules were bispecific [8]. He pursued the evidence and ultimately, in collaboration with Paul Parren, proved that these molecules were produced by a process now known as Fab-arm exchange (Figure 1A) [10]. The original studies focused on proving that several molecules had one arm specific for one allergen, e.g., mites, and the other arm specific for another allergen e.g., grass [11]. However, given that the recombination process has to be random it is obvious that there would be bispecific molecules with the second arm specific for another antigen that is not related to allergic disease or potentially another allergen from the same source (Figure 1B). We assume that the proportion of these three possible forms will reflect the quantities of specific IgG4 in the circulation as a percentage of total IgG4. Thus, the accurate measurement of specific IgG4 in the serum becomes a significant issue. Today many of the commercial monoclonal antibodies, e.g., dupilumab, are produced in or engineered onto a human IgG4 molecule. Their primary purpose is to bind a relevant epitope and to protect this objective they are in most cases modified genetically at the hinge region so that the two disulfide sites are further apart, which effectively prevents Fab-arm exchange.

## 3. Measurement of Specific IgG4 Antibodies in the Circulation

The measurement of specific IgG4 antibodies to allergens did not become possible for use in clinical investigation until the development of monoclonal antibodies (mAb) to this isotype. The first mAb available in Europe was produced by Jefferis in Birmingham, UK [12]. Those antibodies were used in London to measure specific IgG4 using a radioimmunoprecipitation technique that required both mAb to IgG4 and goat or rabbit polyclonal antibodies to mouse IgG. This presented problems because polyclonal anti-mouse IgG produced in another mammal cross-reacts extensively with human IgG and, in keeping with that, the assay could only be a specialized research protocol. Initial studies documented specific IgG4 to grass, mite and milk allergens [13]. The alternative technique at that time was to use ELISA but that technique had such a bad reputation for measuring IgE to allergens that several authors were reluctant to use it for measuring allergen-specific antibodies of other isotypes. Furthermore, it was initially difficult to quantitate the IgG4 measurements accurately using any technique. This contrasted with the progressive development of accurate quantitative measurements of specific IgE using RAST and the first and second generations of ImmunoCAP. It is the second generation ImmunoCAP modified for assaying IgG4 that is most widely used today [14,15].

## 4. Clinical Results for IgG4 Antibodies Using Modified Protocols on Microchip or High Capacity Platforms

Today there is a large number of publications reporting results for specific IgG4 with either microchip techniques or the modified protocol with ImmunoCAP [15,16]. The results are increasingly given in µg/mL (or mg/L). Surprisingly, there have been several papers published where the units were not correct. This problem has appeared so often that there must be a reason. We do not believe that the ImmunoCAP instrument reports the numerical values incorrectly but that the instructions related to the calculation of the units for specific IgG4 are complex and confusing. The most common error is to present the IgG4 antibody as mg/mL, which can easily give values that are greater than total IgG4 [17,18,19]. In addition, a few articles have been published with values of ng/L; these values are much too low but may be due to a typographical error because mg/L is correct [20,21]. Several of these papers have been changed online to present the correct values. In order to emphasize the difficulty in the calculations, during the work on our first publication using ImmunoCAP 250 to measure specific IgG4 we recruited Jonas Lidholm from Phadia Thermo Fisher as an author to be sure of the correct calculations [15]. To add to the confusion, the values used for the measurement of total serum IgG4 are mg/dL where dL stands for deciliter, which is an unusual unit meaning 100 mL. The reason for emphasizing this is that these values are highly relevant to the calculations of the relationship between specific IgG4 values and either total serum IgG4 or specific IgE to the same allergen. In sera from patients with eosinophilic esophagitis (EoE) the quantities of specific IgG4 can be as high as 100 µg/mL and reach levels where the specific antibodies to cow’s milk proteins collectively represent up to 40% of total IgG4 [15]. In turn those values may be highly relevant to understanding that disease.

Much of the evidence about the clinical relevance of IgG4 antibodies has come from studies on immunotherapy for pollen, venom or inhaled allergens other than pollen. In those studies, it was clear that titers of specific IgG4 rise significantly and may reach levels at which they can inhibit in vitro tests such as the basophil activation test (BAT).

**Figure 2:**
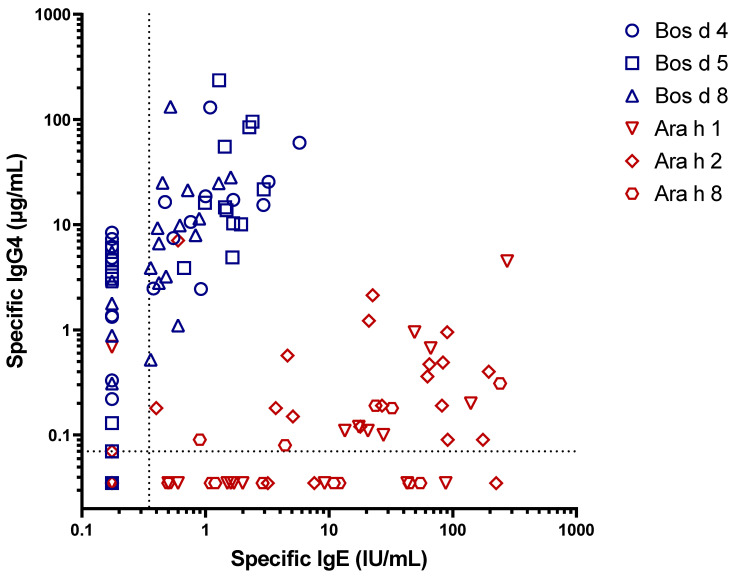
IgE and IgG4 to peanut and milk proteins (Adapted from Wilson et al. [23]).

There have also been studies on IgG4 development in birth cohorts using the Immuno Solid-phase Allergen Chip (ISAC). The results there have shown a wide range but consistently there have been high results for IgG4 to the milk proteins compared with the pollens [22]. However, a limitation of using the microchip for IgG4 is that the results are only semi-quantitative. Our own assays on a birth cohort in 2017 gave striking results using ImmunoCAP to measure both IgG4 and IgE to purified allergens from peanuts and cow’s milk. Among children with detectable IgE antibodies to either of those allergens, the IgG4 antibodies to cow’s milk proteins were dramatically higher than those to peanuts (Figure 2) [23]. Furthermore, we found that in a few sera even ImmunoCAP was underestimating the titer of IgE antibodies to milk because of the high titers of IgG4 antibodies [24]. The problem of high titer IgG4 Ab interfering with IgE measurements can be particularly severe using ELISA or microchip IgE measurements [25].

## 5. The Case for Cat Ownership

Although cats are undoubtedly an excellent source of airborne allergens and fully capable of making children sick with asthma, there is a persisting enigma about the relationship between cat ownership and allergic reactions to cat allergens. When Hesselmar and Bjorksten in Sweden first reported that cat ownership could reduce the risk of a cat allergy, there was, as is normal in science, widespread disbelief [26]. However, when we and others actually took the time to look at our own data, we found the same phenomenon. During the six years prior to 2000, we had been enrolling school children age 11–12 from three socially and geographically different areas of the USA [27]. Among those children, who were in most ways randomly selected, we found a significant inverse relationship between cat ownership and sensitization to cat allergens [28]. That result clearly posed the question about what effect cat ownership had on IgG antibody production and specifically on IgG4 [29]. By that time, i.e., 2000, it had been fully accepted that the switch of immunoglobulin genes to IgG4 was dependent on IL-4 as well as IL-10, which strongly aligned IgG4 with Th2 immunity. For this reason, we used the term “a modified Th2 response” to describe an immune response to a well-recognized allergen that included high titers of specific IgG4 to a known allergen together with low or negative specific IgE [28,30].

There are several factors that are relevant to understanding how or why sensitization to cat allergens could be more common among children living in homes without a cat. Firstly, cats produce large quantities of allergens that remain airborne in such a way that daily exposure is at least ten-fold greater than that to other inhalant allergens. Secondly, the direct measurement of Fel d 1 in dust samples has demonstrated significant quantities of this allergen in public places and schools as well as in homes without a cat [31,32]. Those values are certainly high enough to explain the sensitization of children who have not spent any prolonged time in a house with a cat. Thirdly, the quantities of cat allergens inhaled in a house with a cat may also be sufficient to induce IgG and particularly IgG4 antibodies to cat allergens.

Studies in different environments or with different protocols have given mixed results on the effects of cat ownership. However, it is clear that the effect of cat ownership on the rest of the community depends on the prevalence of cat ownership in the community. If cats are present in less than 10% of homes, then cat ownership is likely to be a significant risk factor for sensitization [33]. In complete contrast, in a community where ≥ 60% of houses have cats, the exposure of children living in the minority of houses without a cat may also be high enough to induce a tolerance. The relevance of the prevalence of cats in the community was spelled out by Svanes et al. who analyzed several studies and concluded that the inverse relationship between cat ownership and sensitization to cat allergens was most likely to be seen when 20–40% of the houses had a cat [33]. Perhaps the most extreme example comes from New Zealand where >65% of homes have a cat. In an ISAAC-based cohort study on asthma in childhood together with Dr. Crane and Dr. Wickens, we confirmed what had already been seen in the Dunedin cohort: that sensitization to cat allergens was much less relevant than mite sensitization [34,35]. Among the 112 asthmatic children, 55 were living in a house with a cat and, of those, 34 were sensitized to dust mite allergens but not sensitized to cat allergens [35]. Thus, there can be no doubt that a high exposure (i.e., cat ownership) can either act to sensitize to cat allergens or to induce tolerance. Strikingly, cat ownership was negatively associated with cat sensitization but had no effect on the prevalence or titer of IgE antibodies to mites. From our own data in Sweden as well as from two birth cohorts in the USA, it is clear that specific IgG4 antibodies to cats are an important part of the immune response to cat allergens in children who do not have symptoms related to cat exposure (Table 1) [36].

## 6. Food Allergy and Specific IgG4 Antibodies to Food Allergens

Over the 35 years that Peter Heymann has been in charge of the pediatric allergy clinic at the University of Virginia, three different forms of food allergy have increased. In two cases, they have become apparent for the first time. These increases between 1990 and the present should be compared with the history of seasonal allergic rhinitis, which increased steadily from 1870 to its peak around 1970 while asthma in childhood increased dramatically from 1960 to around 2000 [38]. The three forms of food allergy that are now common in Virginia are (i) immediate hypersensitivity to peanuts, (ii) delayed anaphylaxis to mammalian meat, also called the alpha-gal syndrome (AGS) and (iii) eosinophilic esophagitis (EoE) [15,39]. The first two are characterized by high titer IgE antibodies and are considered to be IgE-mediated. By contrast, EoE is not mediated by specific IgE antibodies. A peanut allergy was always present but started to increase in 1990 and may still be increasing today [40]. The alpha-gal syndrome was first identified in the USA in 2008 and has been increasing northwards with the expanding range of the Lone Star tick [41,42]. However, recent evidence suggests that we may expect a decrease in the south because of the relentless spread of fire ants, which are a major predator of these ticks [43]. The third disease, EoE, was first recognized as a form of food allergy by Kelly et al. in 1995 and has now increased to the level where many academic centers have special clinics for both pediatric and adult cases [44]. EoE is characterized by an eosinophil rich inflammation of the esophagus that can progress to fibrosis and stricture formation [45]. The patients are generally not aware of immediate symptoms after eating food but clearly respond to the diet and can relapse over weeks with the reintroduction of the food. It is unusual for these patients to have high titer IgE antibodies to the relevant foods, which many studies have shown to be dominantly cow’s milk and wheat [46]. To give proper credit, Sampson first described the response to the diet and approximately 10 years ago Spergel said “it does not matter what diet you choose, but the first element must be cow’s milk avoidance” [44,46]. In keeping with that, Dr. Erwin in Columbus, Ohio carried out a trial of six weeks’ avoidance of cow’s milk proteins. In that trial the children who had low levels of specific IgE to milk (i.e., between 0.1 and 1.0 IU/mL) responded very well [47]. However, those children were not aware of being allergic to milk and the disease did not improve with anti-IgE treatment.

**Figure 3:**
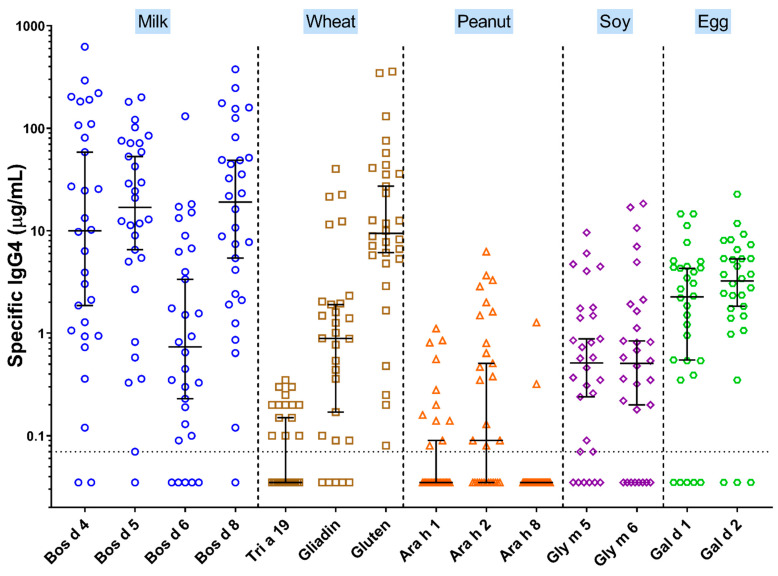
IgG4 to multiple allergens in serum from 30 children with EoE (Adapted from Schuyler et al. [15]).

In 2014, Dr. Clayton working with Kathy Peterson in the adult gastroenterology group in University of Utah School of Medicine in Salt Lake City reported striking evidence that adult patients with EoE have elevated serum food-specific IgG4 antibodies [48]. In addition, they reported the presence of easily visualized extracellular deposits of IgG4 in the wall of the esophagus. The findings related to serum IgG4 antibodies in adults with EoE were confirmed by Wright and Dellon while the presence of IgG4 deposits has been confirmed by several groups [49]. Our own focus on pediatric cases has been on serum IgG4 antibodies and the results are truly astonishing [15,23] (Figure 3). Using an ImmunoCAP system (Thermo-fisher/Phadia U.S., Portage, MI, USA) modified to assay IgG4 antibodies, we found both a high prevalence and very high titers of IgG4 antibodies to the cow’s milk proteins alpha-lactalbumin (Bos d 4), beta-lactoglobulin (Bos d 5) and casein (Bos d 8) as well as to gluten from wheat [15,23]. In many cases specific IgG4 was ≥10 µg/mL and in a few cases the sum of IgG4 specific for Bos d 4, Bos d 5 and Bos d 8 was ≥100 µg/mL.

The association of tissue deposits of IgG4 and elevated total serum IgG4 has been seen before in the so called “IgG4-related diseases”. However, in those diseases, the specificity of IgG4 antibodies is generally not known. The titers of specific IgG4 to milk and wheat proteins in children with EoE are the highest values for IgG4 to any antigen that we are aware of. Indeed, a few sera from patients with EoE have collective quantities of specific IgG4 as high as 40% of total IgG4 [15]. We would emphasize that it is essential to have accurate measurements of specific antibodies to evaluate ratios of specific antibodies to total quantities of an isotype. The fact that patients with EoE can have ≥100 µg/mL of specific IgG4 to milk proteins and that the total IgG4 in many sera is ≤1 mg/mL makes it obvious that the accuracy of these measurements is essential (Figure 3, Table 1).

The presence of high titers of specific IgG4 to Bos d 4, Bos d 5 and/or Bos d 8 in patients who have extracellular deposits of IgG4 in the esophagus raises several questions. The primary questions should be why do these patients make such striking antibody responses to these important food sources and is there a reason why this has increased? The secondary question is why does IgG4 accumulate in the local tissue? Antibodies of this isotype have a molecular weight of ~180,000 and should rapidly diffuse out of local tissue or away from a plasma cell unless they are bound in some way. However, as we discussed earlier, IgG4 antibodies in general do not form complexes or bind to most Fc receptors. Preliminary data suggest that milk and or wheat proteins can also accumulate in the areas of the esophagus where deposits of IgG4 are present.

**Figure 4:**
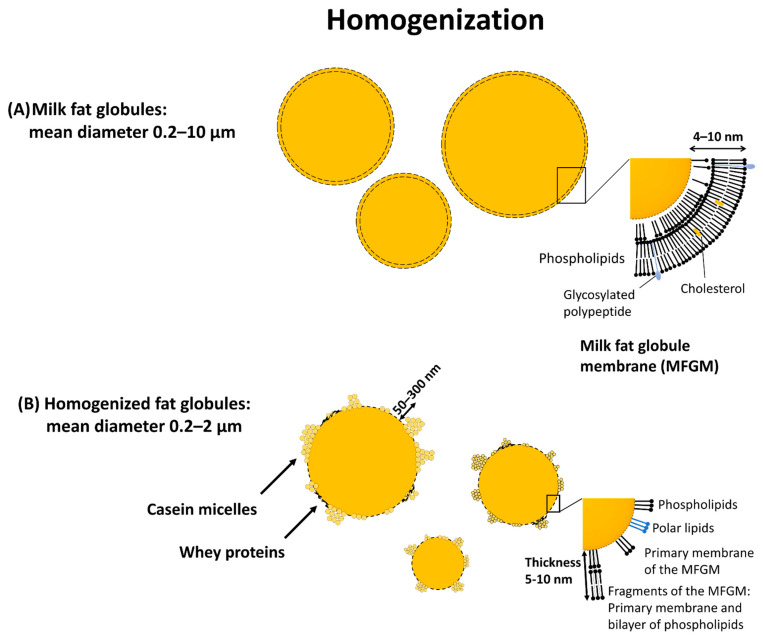
High pressure homogenization as part of the processing of ultra-heated milk Adapted with permission from Vignolles et al. [51]. Copyright 2007 EDPSciences.

The accumulation of IgG4 molecules locally in the esophagus could theoretically occur as a result of several processes:

(1) It has been suggested that antibodies of this isotype have a tendency to aggregate if present in high concentrations [50].
(2) Bivalent IgG4 antibodies coming out of a plasma cell could form complexes if they encounter milk proteins or other relevant antigens close to their site of production. A few of the bispecific molecules of IgG4 could be of the A1 + A2 type (See Figure 1B) potentially cross-linking molecules such as Bos d 4 and Bos d 5.
(3) While most of the protein in the whey fraction of milk is present as single molecules, the same is not true of casein molecules, which form micelles that can reach large sizes. It is this characteristic of caseins that leads them to form “curds” and thus in most cheeses they are the major component. The molecules of casein also adhere strongly to the disrupted smaller fat particles in homogenized milk [51] (Figure 4). If multiple caseins and other milk proteins adhere to droplets as small as 40 nm in diameter, they could easily penetrate the walls of the esophagus and act as a potent immunogen. In addition, these small particles could act as a nidus for the local accumulation of IgG4 molecules.

It is important to recognize that milk protein consumption is measured in grams per day. This is thousands of times greater than any inhalant allergen. It has been reported by several groups that treatment with oral immunotherapy (OIT) in patients with immediate reactions to peanuts, milk or egg can give rise to an EoE-like condition [52]. There are two relevant facts here: firstly, we and others have recorded increases in serum IgG4 antibodies during OIT with food allergens. In collaboration with a group in San Paulo, Brazil, we reported to the American Academy of Allergy, Asthma and Immunology (AAAAI) meeting in 2019 that the subjects receiving milk OIT had major increases in specific IgG4 to Bos d 4, Bos d 5 and Bos d 8 [53]. Secondly, this same Brazilian group have reported that many of the patients on milk OIT had increased esophageal eosinophils before they started being treated with oral milk proteins [54].

## 7. Why Has This Specific IgG4-Related Disease Increased So Dramatically?

When the senior author of this manuscript was a student at St. Thomas’s Hospital in the 1960s, Norman Barrett was still carrying out ward rounds. At that time, he apparently was not and certainly we students were not aware of an eosinophilic inflammatory disease of the esophagus that could give rise to severe symptoms or obstruction. In keeping with that, neither PWH nor TP-M were aware of cases of EoE in the clinics or emergency departments of the University of Virginia in the 1980s. Thus, the real question is what has happened that could lead to the emergence of this disease in the 1990s and the increase since then? One obvious possibility is that changes in the processing of food lead to forms of milk and/or wheat that are more immunogenic. Two aspects of the processing of milk could be relevant; they are ultra-heat treatment and homogenization. Until recently, bottled milk delivered to the door in London had been pasteurized at 75 °C and was not homogenized. By contrast, greater than 90% of cow’s milk sold in the USA today has been heated to 135–140 °C as well as being homogenized. In fact, there is no such thing as non-homogenized ultra-heat-treated milk because the fat in non-homogenized milk accumulates at the surface as cream but over time the cream layer changes to a form more like brittle rubber. The homogenization of milk started as early as the 1940s and has not only become more common but has also become more efficient. The details of this process of “high-pressure” may well be relevant to understanding the increases in EoE [51,55]. We have focused on the relevance of changes in the processing of cow’s milk for several reasons. Firstly, this is the most commonly identified allergen contributing clinically to EoE and in many studies this allergen source appears to be relevant to 80% or more of the patients. Secondly, there have been major changes in the processing of cow’s milk that could be relevant to the disease process. These changes include increased pressure used for homogenization and increased temperature used for “pasteurization”. Thirdly, in several studies on dietary treatment, the avoidance of mammalian dairy products alone has resulted in a remission of 30% or more of the cases. We are well aware that other allergen sources, e.g., wheat and egg, can contribute to both specific IgG4 and the clinical syndrome but we know less about the processing of these foods and particularly less about the effects of processing on the form in which they are most often eaten.

The primary objective of homogenization is to decrease the size of the fat droplets so that the milk becomes a stable emulsion. The average change in the diameter of these droplets is from 3 microns to 300 nanometers. This change results in a thousand-fold increase in the number of fat droplets and a tenfold increase in the total surface area. The change in the surface area leads to a profound disruption of the surface of the droplets, which in turn encourages the adherence of milk proteins to the surface of the particles (Figure 4). The striking thing is that these droplets post-homogenization are very similar to the optimal characteristics for oral immunization [56]. Thus, changes in the processing of milk may explain why the IgG4 antibody responses to those particular proteins are so impressive. On the other hand, it is likely that there have been other changes to our lifestyle that could have helped to initiate damage to the epithelium of the esophagus. The most extensively studied and relevant changes in lifestyle are increases in exposure to detergents in both water and dishwashing fluid, for example. These detergents can disrupt epithelial cell connections at very low doses [57]. In keeping with this, Rothenberg and his colleagues in Cincinnati have identified correlations of EoE with two genes that can influence the permeability of the esophageal epithelium [58].

## 8. Summary and Conclusions

Three forms of food allergy have dramatically changed the clinical practice of allergic disease in the United States. In addition, they have provided exceptional opportunities for research into both the mechanisms of the disease and the epidemiology of changes in the prevalence. Although two of the syndromes are characterized by anaphylaxis, which can become severe, the timing in relation to eating is completely different. The speed with which allergic children can react to peanut exposure is well-known. In the alpha-gal syndrome (AGS), although reactions are generally delayed for at least 2 h, once started they can become severe rapidly [59]. Finally, EoE is the most distinct; in general, the patients have no awareness of being “allergic” to the main foods that cause the disease and consequently continue to consume grams of cow’s milk proteins per day (Box 1). After being on an avoidance diet, patients with EoE can wait days to weeks after reintroduction before their symptoms relapse.

Box 1. **Three forms of food allergy that are distinct clinically, histopathologically and in the apparent causes of increased prevalence.**

IgEIgG4 to IgE RatioI. Peanut anaphylaxis: rapid allergic reactions occurring with peanuts or other foods. High titer IgE to defined proteins from the relevant foods. Increase: decrease in the skin barrier possibly related to detergents; a strong correlation with atopic dermatitis.High
**~1:1**
II. Eosinophilic esophagitis (EoE): delayed onset of symptoms (i.e., days); low IgE and high or very high IgG4 to cow’s milk and/or wheat proteins. Increase: food processing and damage to the esophageal barrier.Low or negative
**~500:1**
III. Delayed anaphylaxis to mammalian meat: alpha-gal syndrome (AGS) reactions delayed in onset but can be severe. Increase: increased bites from Lone Star ticks; in the USA, associated with an extraordinary increase in the deer population close to houses.Low to high
**~1:1**

Studying IgG4 antibody responses, the differences between common inhalants such as pollen or dust mite when compared with cat allergens can best be explained by the quantity of exposure. For most inhalant allergens, exposure is measured in ng/day; indeed, Marsh estimated that exposure to specific pollen allergens was ~1 µg/year [60]. In keeping with that, increasing the exposure during subcutaneous or oral IT will increase serum IgG4 consistently. The estimates of inhaled exposure to cat allergens are in µg/day for children living in a house with a cat and the average levels of specific IgG4 are as much as ten-fold higher than those for the other inhalants. Thus, it is not surprising that continuous or daily exposure to cats can induce a form of tolerance with high levels of IgG4. The important thing about food allergens is that many people living on unprocessed foods appear to make low levels of IgG4 antibodies to commonly consumed foods. For example, levels of IgG4 to each of Bos d 4, Bos d 5 and Bos d 8 were lower among Amish than Hutterite children [61]. Thus, although the quantities of milk proteins eaten daily are measured in grams, the quantities of milk-specific IgG4 seen in EoE as well as in a proportion of the teenage children in the VIVA birth cohort should not be considered “normal”. Given that many children with EoE continue to consume milk, it is perhaps not surprising that IgG4 antibodies accumulate in the area where IgG4 antibodies are being produced. The question is whether these ‘complexes’ can have an inflammatory effect related to eosinophils. The alternative explanation is that IgG4 production, eosinophil recruitment and the relative suppression of IgE antibodies all reflect the T-cells induced by exposure to processed milk or wheat.

In the United States, the increase in AGS is almost certainly related to an increased exposure to the Lone Star tick *Amblyomma americanum*. Tick bites can give rise to high levels of IgE specific for the oligosaccharide galactose alpha-1,3-galactose (alpha-gal). The production of antibodies to this oligosaccharide can occur because the gene for the relevant alpha-1,3-galactosyltransferase became inactivated in primates around 20 million years ago [62,63]. Although the tick is moving north, at the same time it is being controlled in the south by the relentless advance of its principal predator, the red imported fire ant [41,43]. Interestingly, the impact of glycolipids/glycoproteins and other enhancers in tick saliva, which are such a potent stimulus to IgE production, are not a significant inducer of specific IgG4 either for alpha-gal or tick antigens (see Table 1 and Box 1). The true implication of these levels of specific IgG4 relative to the understanding of the nature of the stimulus to IgE is not clear. However, the low levels of IgG4 in both the antibody response to peanuts and tick bites represent yet another aspect of these different forms of food allergy that should be further studied.

### Conclusions

We have described two situations where the increased levels of specific IgG4 to an allergen are associated with a difference in an allergic disease. Firstly, many children living in a house with a cat produce relatively large quantities of specific IgG4 to cat allergens together with low levels of specific IgE. This form of response is not related to asthma and can lead to a situation where owning a cat decreases your child’s risk of having asthma related to cat allergens [26,28,33,36]. In the second situation, high levels of specific IgG4 to Bos d 4, Bos d 5 and Bos d 8 are now relatively common and are strongly associated with EoE. Luckily, at present, among the children with high levels of specific IgG4, i.e., ≥10 µg/mL, only a small percent develop symptoms of EoE. However, there is a good case for considering that changes in the processing of milk including highly efficient high pressure homogenization have made major contributions to this immune response and indirectly to the damage to the esophagus [51,55]. Interestingly, there are many ways that this specific IgG4-related disease in the esophagus is similar to other IgG4-related diseases. The difference is that we are well aware that foods and particularly cow’s milk and wheat are the major cause. In our view, it is very likely that both IgE-mediated forms of food allergy and also EoE will be shown to be due to changes in human behavior/lifestyle.

## Figures and Tables

**Table 1:** IgG4 antibody titers to cat and milk antigens in children with a high exposure compared with IgG4 antibodies to dust mites and alpha-gal (Unpublished data and data adapted from Schuyler et al. ^ϯ^ [15] and Wilson et al. * [37]).

		Prevalence of Different Levels of IgG4		
		≥0.07 µg/mL	≥1 µg/mL	≥10 µg/mL
Cat dander	Owners	72/83	19/83	0/83
	Non-owners	85/108	8/108	0/108
Fel d 1	Owners	51/83	12/83	0/83
	Non-owners	24/83	0/83	0/83
Milk ^ϯ^	Bos d 4	146/210	87/210	21/210
	Bos d 5	184/210	135/210	40/210
	Bos d 8	204/210	114/210	23/210
Dust mite	*D. pteronyssinus*	28/38	0/38	0/38
	Der p 1	22/38	0/38	0/38
	Der p 2	8/38	0/38	0/38
	Der p 23	1/38	0/38	0/38
Alpha-gal *	Alpha-gal-HSA	9/63	0/63	0/63
